# Supplementary material for: Care by Midwives, Obstetricians, and Dietitians for Pregnant Women Following a Strict Plant-Based Diet: A Cross-Sectional Study
Source: Nutrients. 2021 Jul 13;13(7):2394. doi: 10.3390/nu13072394 (PMC8308805; doi:10.3390/nu13072394)
Supplement: Supplementary file 1 [file nutrients-13-02394-s001.zip › Supporting information S1 questionnaires midwives and obstetricians.pdf]

Supporting information S1 – Questionnaire midwifes and obstetricians.

1. In which area of the Netherlands do you work?
    a. Groningen
    b. Drenthe
    c. Flevoland
    d. Noord-Holland
    e. Overijssel
    f. Zuid-Holland
    g. Utrecht
    h. Gelderland
    i. Zeeland
    j. Noord-Brabant
    k. Limburg
2. How many other midwives work at your practice? *
    a. One-man practice
    b. Practice with 2-5 midwives
    c. Practice with 6+ midwives
3. What is the name of your hospital?**
4. What is your gender?
    a. Male
    b. Female
5. What is your age?
    a. <30
    b. 31-40
    c. 41-50
    d. >50
6. Do you ask pregnant women about diet at the first prenatal checkup
    a. Always, it is a mandatory box
    b. Most of the time, entered in the file
    c. Sometimes
    d. Almost never
    e. These women tell this themselves
    f. Other

Important note: A vegan diet is a diet excludes all animal products, e.g. meat, fish, eggs or dairy products. The following questions are about a **vegan** diet, not a vegetarian diet.

7. Is there a protocol or agreement on how to act when a pregnant woman is on a vegan diet?
    a. Yes
    b. No
    c. I don't know if there is a protocol
8. Do you think your education about nutrition in your program was sufficient?
    a. Yes, I think I have learned enough
    b. Yes, but I think it was insufficient
    c. No, I barely learned about nutrition
9. Have you followed an additional course about nutrition?
    a. Yes
    b. No

i. If yes, was a vegan diet included in this additional course?
   a) Yes
   b) No

10. Who do you think is responsible for advising pregnant women on a vegan diet about nutrition and lifestyle?
    b. Midwife
    c. Nurse
    d. Dietician
    e. Voedingscentrum
    f. Government
    g. General practitioner
    h. Patient herself
    i. Other
11. Did you do a consultation with a pregnant woman on a vegan diet in the last year?
    a. Yes, >10 times
    b. Yes, 5-10 times
    c. Yes, 0-5 times
    d. No
    e. I don’t know
12. Do you think you have sufficient knowledge to advise a pregnant woman on a vegan diet about her diet in her pregnancy?
    a. Yes
    b. No
    c. I don’t know
13. Do you implement additional policies as soon as you know that a pregnant woman is on a vegan diet? And if so, what does this additional policy entail?
    a. Yes
    b. No
       i. If Yes…
          a) Refer to a dietician
          b) Blood test of vitamin status
          c) Checking vitamin B12 in blood
          d) Checking iron in blood
          e) Checking hemoglobin in blood
          f) Giving supplements
          g) Giving advice
          h) Ask about own knowledge
          i) Other…
14. Do you think pregnant women on a vegan diet are more likely to be nutritional insufficient compared to women with a regular diet?
    b. Yes
    c. No
       i. If Yes…
          a) Carbohydrates
          b) Protein
          c) Fats
          d) Omega
          e) Saturated fats
          f) Calcium
          g) Magnesium

h) Phosphorus
i) Potassium
j) Iodine
k) Iron
l) Selenium
m) Zinc
n) Vitamin A
o) Vitamin B1
p) Vitamin B2
q) Vitamin B6
r) Vitamin B12
s) Folic acid
t) Vitamin C
u) Other…

15. Who do you think is responsible for advising pregnant woman on a vegan diet regarding breastfeeding/vegan formula feeding?
    a. Midwife
    b. Nurse
    c. Dietician
    d. Voedingscentrum
    e. Government
    f. General practitioner
    g. Well-baby clinic
    h. Patient herself
    i. Lactation consultant
    j. Maternity care
    k. Other…
16. When do you think women on a vegan diet should receive information about their diet in childbirth with regards to breastfeeding / artificial feeding?
    a. During their pregnancy
    b. Shortly after labor
    c. Other…
17. Do you think lactating women on a vegan diet should consume extra nutrients or take extra supplements?
    d. Yes, namely…
    e. Yes, but I do not know which
    f. No additional measures are needed
    g. I do not know
18. Which of the following options would you prefer to improve attention for nutrition during pregnancy and during breastfeeding for women on a vegan diet?
    a. Every midwife/obstetrican/dietician should have the knowledge to advise these women
    b. One midwife/obstetrican/dietician in each region should have the knowledge to advise these women
    c. The midwife/obstetrican is signaling and sends these women to a dietician
    d. The midwife/obstetrican/dietician is signaling and sends these women to a website/flyer/helpline
    e. Nothing has to be changed
    f. Other…

19. Do you want to learn more about the vegan diet during pregnancy? If Yes, what form would you prefer?
    a. One-day course
    b. Online course
    c. Flyer
    d. Protocol
    e. No
    f. Other…

* This question was only included in the questionnaire for midwives.

** This question was only included in the questionnaire for obstetricians.
