# Supplementary material for: Care by Midwives, Obstetricians, and Dietitians for Pregnant Women Following a Strict Plant-Based Diet: A Cross-Sectional Study
Source: Nutrients. 2021 Jul 13;13(7):2394. doi: 10.3390/nu13072394 (PMC8308805; doi:10.3390/nu13072394)
Supplement: Supplementary file 1 [file nutrients-13-02394-s001.zip › Supporting information S2 questionnaires dieticians.pdf]

Supporting information S2 – Questionnaire dieticians.

1. What is your age?
    a. <30
    b. 31-40
    c. 41-50
    d. >50
2. How many years do you work as a dietician?
3. Describe your job as a dietician, multiple answers are possible:
    a. Dietician in a first-line dietician practice
    b. Dietician active in nursing or home care
    c. Dietician active in education or training institutions
    d. Dietician active in mental healthcare
    e. Dietician working with (mentally) disabled people
    f. Dietician active in the rehabilitation care
    g. Dietician active in the youth assistance
    h. Dietician active in the addiction care
    i. Lifestyle advisor
    j. Sport dietician
    k. Business dietician
    l. School dietician
    m. Research dietician
    n. Other…
4. In which area in the Netherlands do you work?
    a. Groningen
    b. Drenthe
    c. Flevoland
    d. Noord-Holland
    e. Overijssel
    f. Zuid-Holland
    g. Utrecht
    h. Gelderland
    i. Zeeland
    j. Noord-Brabant
    k. Limburg

Important note: A vegan diet is a diet excludes all animal products, e.g. meat, fish, eggs or dairy products. The following questions are about a **vegan** diet, not a vegetarian diet.

5. Did you receive any education regarding pregnancy and a vegan diet in your program?
    a. Yes
    b. No
    c. No, but I did learn about a vegetarian diet during pregnancy
6. Have you followed an additional course about nutrition during pregnancy?
    a. Yes, this included advice about a vegan diet during pregnancy
    b. Yes, this included **only** advice about a vegetarian diet during pregnancy
    c. Yes, but this did **not** cover a vegetarian or a vegan diet during pregnancy
    d. No
7. Statement: I think I have sufficient knowledge to advise pregnant women on a vegan diet during pregnancy:

    a. I agree
    b. I don’t know
    c. I disagree
8. Have you seen a pregnant woman on a vegan diet in the last year?
    a. Yes, >10 times
    b. Yes, 5-10 times
    c. Yes, 0-4 times
    d. No
9. Were these women referred by a professional?
    a. No, these women come on their own initiative
    b. Yes, these women were referred by a obstetrican
    c. Yes, these women were referred by a midwife
    d. Yes, these women were referred by another dietician
    e. Yes, these women were referred by a general practitioner
    f. Yes, these women were referred by information of the website of the *Voedingscentrum*
    g. Other…
10. When you want to look up information about a vegan diet in pregnancy, where would you search?
    a. *Voedingscentrum*
    b. The Dutch magazine for nutrition and dietetics
    c. Colleagues
    d. Other…
11. When do you think women with a vegan diet should get information or advise?
    a. When they have an active wish to have children
    b. During their pregnancy
    c. It is not necessary
12. Do you want to learn more about a vegan diet during pregnancy?
    a. Yes
    b. No
13. What type of education would you prefer to raise the awareness of dieticians for pregnant women on a vegan diet?
    a. A lecture during congresses for healthcare professionals
    b. A course
    c. A national protocol
    d. I do not think this is necessary
